# Data‐driven thresholds for standardized classification of severe Alzheimer's disease neuropathology using digital neuropathology

**DOI:** 10.1111/bpa.70102

**Published:** 2026-04-26

**Authors:** Ryan K. Shahidehpour, Allison M. Neltner, Mitchell A. Klusty, Cole Corbett, Angelique D. Gonzalez, David A. Gutman, David W. Fardo, Adam D. Bachstetter, Cody Bumgardner, Margaret E. Flanagan, Peter T. Nelson

**Affiliations:** ^1^ Sanders‐Brown Center on Aging University of Kentucky Lexington Kentucky USA; ^2^ Department of Neuroscience University of Kentucky Lexington Kentucky USA; ^3^ Institute for Biomedical Informatics University of Kentucky Lexington Kentucky USA; ^4^ Glenn Biggs Institute for Alzheimer's and Neurodegenerative Diseases University of Texas Health Science Center at San Antonio San Antonio Texas USA; ^5^ Department of Pathology Emory University School of Medicine Atlanta Georgia USA; ^6^ Spinal Cord and Brain Injury Research Center University of Kentucky Lexington Kentucky USA; ^7^ Department of Pathology and Laboratory Medicine University of Kentucky Lexington Kentucky USA; ^8^ Department of Pathology and Laboratory Medicine University of Texas Health Science Center at San Antonio San Antonio Texas USA

**Keywords:** artificial intelligence, HALO, inter‐rater, PHF1, ScanScope, tauopathy

## Abstract

Alzheimer's disease neuropathological changes (ADNC)—operationalized with semi‐quantitative parameters—represent the consensus‐based gold standard for diagnostic evaluation of disease severity. Although useful, ADNC diagnostic frameworks have limitations, particularly in advanced disease stages where pathological severity varies widely within a given diagnostic category. Further, some individuals lacking cognitive impairment are inappropriately categorized as having severe ADNC. In this study, quantitative pathology metrics and alternative tissue sampling schemes were integrated with data about premortem cognitive status, in order to derive clinically informed neuropathologic diagnostic thresholds. Specific goals of the current study were to generate data‐driven, standardized diagnostic cut‐points, with the most severe stages of ADNC having consistent implications: Braak neurofibrillary tangle (NFT) stage V cases being always impaired (MCI or demented) and Braak NFT stage VI cases being always demented. Utilizing whole‐slide imaging and AI‐based image analysis, object‐based (NFT counts) and pixel‐based (phosphorylated tau [pTau] burden) quantifications were compared across neocortical regions in three subsamples of cases from the University of Kentucky ADRC autopsy cohort (*n* = 329, all with clinical evaluations within 2 years of death). We also compared between HALO‐ and Aperio‐based platform results, and between AT8 and PHF‐1 pTau antibodies. Applying refined thresholds enabled reclassification of cases previously misaligned with their digitally determined appropriate status: 17% of cases were thus reclassified. The use of commercially available software, standardized classifier architectures, and interoperable analysis pipelines facilitated scalable and reproducible digital quantification. Cross‐institutional validation at University of Texas San Antonio, with the same algorithms applied in both research centers, confirmed near‐perfect agreement of pathology counts, underscoring shareability and the feasibility of harmonized digital workflows for collaborative research and diagnostic purposes. These findings support the integration of quantitative digital pathology into standard neuropathological protocols and provide a scalable model for future multi‐site studies. Enabling comparisons of analytical platforms, pTau antibodies, and anatomical sampling strategies, an updated workflow demonstrated high reproducibility and consistent clinical‐pathological correlations.

## Graphical abstract

We generated digital pathologic algorithms (AI‐based modules that can be freely shared) to: optimize the sampling (ROI) for diagnosis of ADNC‐related tau pathology; compare/transition between Aperio and HALO platforms; and rationalize severe Braak NFT staging, so that all Braak‐VI cases had dementia, and all Braak‐V cases had MCI or dementia.

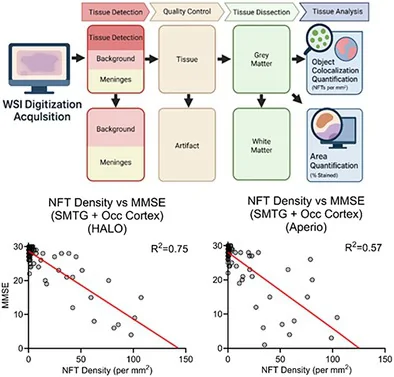

## 1 INTRODUCTION

The hallmarks of Alzheimer's disease neuropathologic change (ADNC) are β‐amyloid peptide (Aβ) deposition, neuritic amyloid plaques, and neurofibrillary tangles (NFTs), each categorized using semiquantitative metrics, according to the National Institute on Aging–Alzheimer's Association (NIA‐AA) consensus‐based guidelines [1, 2]. Of the ADNC pathological hallmarks, NFTs (intraneuronal inclusions composed of phosphorylated tau, or pTau) have the strongest association with cognitive impairment [3]. Braak NFT staging constitutes a highly ordinal scale (with seven stages, ranging from stage 0 to stage VI), indexing the spatial distribution of NFTs in the brain [4, 5, 6, 7, 8]. Braak NFT staging gauges the stereotypic expansion of pTau pathology from the transentorhinal cortex in isolation (Braak NFT stage I) through limbic regions and ultimately into many neocortical regions in Braak NFT stage VI. In contrast to earlier stages, Braak NFT stages IV through VI are often accompanied by measurable clinical decline [9, 10, 11]. The methodology of Braak NFT staging has evolved from the use of silver stains to pTau immunohistochemistry, with the protocol described by Alafuzoff et al., now serving as the standard for Braak NFT staging [8]. While Braak NFT staging remains a valuable tool for describing (indeed, defining) ADNC severity, individuals assigned to the same Braak NFT stage may exhibit widely varying levels of cognitive function and pathological burden [11, 12, 13]. This imperfect clinicopathological correlation decreases the interpretive utility of Braak NFT staging. The goal of the current study is to tie the pathologic diagnosis more closely to the severity of clinical impairment and also to standardize the use of digital pathology for the purposes of diagnosing ADNC.

Several other limitations of Braak NFT staging have become increasingly apparent in the modern era of digital and quantitative neuropathology. The system of Braak NFT staging is inherently semi‐quantitative, describing the anatomical extent of NFT deposition but not its absolute burden. As a result, two cases with identical Braak NFT staging may differ substantially in pTau pathologic load [14, 15]. Additional variability arises from differences in staining protocols, antibody epitopes, and imaging platforms, all of which may influence the threshold for what constitutes “positive” pathology and ultimately how patients are diagnostically categorized [16]. To help address these limitations, several research groups and consortia have harmonized pTau immunohistochemistry protocols, established standardized regional sampling templates, and introduced complementary criteria to enhance reproducibility [8, 17, 18, 19, 20]. Further, an increasing number of neuropathologists have augmented traditional Braak NFT staging with digital quantification of pTau pathology [14, 21, 22, 23, 24, 25, 26].

The increasing adoption of digital pathologic methods represents a shift from largely semi‐quantitative, anatomically dichotomized staging toward more rigorously quantitative metrics of pathological severity to enable improved alignment between neuropathological assessment and measurable cognitive outcomes. However, to date, digital pathological methods have not been leveraged for data‐driven refinement of the existing semiquantitative metrics. More specifically, no quantitative method currently exists to delineate between stages of severe ADNC as set forth by the NIA‐AA guidelines, based on a developmental model that incorporates digital pathology and clinical data.

Digital pathology is transforming traditional, observer‐dependent evaluations into a more quantitative discipline by digitizing tissue slides and enabling advanced computational analyses [27, 28, 29, 30, 31, 32, 33]. Central to this transformation is the integration of artificial intelligence (AI), particularly machine learning (ML) and deep learning (DL), which have become highly useful for modern pathological workflows. AI algorithms now automate tasks such as image segmentation, feature extraction, and classification, thereby enhancing both diagnostic accuracy and operational efficiency [34, 35, 36, 37, 38]. For neuropathologists, tools such as PathoFusion utilize convolutional neural networks to analyze whole‐slide images (WSIs), detecting subtle but quantifiable histomorphologic features relevant to neuropathology [38, 39, 40, 41, 42, 43]. In addition to identification and quantification of pathologic phenomena related to neurodegenerative diseases, these systems also support tumor grading and multimodal integration with neuroimaging and genomic data for more personalized diagnostics [37, 40, 44, 45, 46, 47, 48].

Recent developments in AI‐driven tools and commercial platforms such as HALO, Aperio, and QuPath now enable high‐throughput, pixel‐level segmentation of pathology across entire slides [49, 50, 51, 52]. Deep learning architectures including U‐Net, SegNet, and fully convolutional networks (FCNs) produce robust metrics for pTau burden, pathology density, and spatial clustering, which together provide robust, comprehensive, and reproducible profiles (quantitative and qualitative) of disease‐related pathologies [53, 54, 55, 56, 57].

The University of Kentucky Alzheimer's Disease Research Center (UK‐ADRC) has incorporated digital pathological techniques since 2012 as part of the routine evaluation of all autopsies, following a published workflow [9, 22]. Through early implementation of WSI and computational analysis, the UK‐ADRC has leveraged image‐derived metrics as part of routine neuropathologic evaluation, facilitating a shift toward integrating quantitative WSI‐based pathologic readouts [22, 58, 59]. In addition to a number of relevant research papers that have resulted from these resources [59, 60, 61, 62, 63, 64, 65, 66], the virtual biobank has facilitated diagnostic pathology at the UK‐ADRC and has been useful for building a federated digital slide archive. However, broader adoption across institutions remains limited by the lack of infrastructure for sharing WSIs and analysis pipelines. Furthermore, the widely used Aperio‐based software for digital analyses is in the process of being phased out by the manufacturer.

To overcome these barriers, we generated, trained, and tested commercially available HALO digital pathology software to enable cross‐institutional sharing of image data and analysis modules. Standardized pipelines, including trained tissue classifiers, object detection algorithms, and analysis parameters were shared with the University of Texas Health Science Center at San Antonio (UTHSA) ADRC, enabling standardized quantification of pTau pathology across sites. This work reflects a broader evolution in the field, from anatomy‐based, semi‐quantitative staging, toward a more quantitative (clinical and pathological) data‐driven system, and underscores the critical role of digital neuropathology in modernizing ADNC assessment [22, 67, 68]. An overarching goal of this study was to develop standardized, quantitative, shareable, and clinically relevant thresholds for distinguishing Braak NFT stages IV–VI, informed by both digital pathology metrics and antemortem cognitive data.

## 2 METHODS

### 2.1 Research participants, subsamples, inclusion, and exclusion criteria

Samples were acquired from the UK‐ADRC autopsy cohort, a community‐based cohort drawing from the Lexington, Kentucky area. The cohort's demographics, recruitment strategies, and other pertinent details have been previously described [69, 70, 71]. The large majority of included participants were recruited into the study while neurologically normal and followed longitudinally to autopsy. Cases included in the study were characterized based on clinical presentation and cognitive abilities (i.e., “normal,” “impaired,” “demented”) at an ADRC consensus conference that included primary input by clinicians, as previously described [72, 73, 74, 75, 76, 77].

Three subsamples were derived from the overall UK‐ADRC cohort, to support training, validation, and cross‐center standardization analyses. Cohort 1 (*n* = 81) comprised “pure” ADNC cases, specifically excluding individuals with coexisting pathologies such as dementia with Lewy bodies (DLB), limbic‐predominant age‐related TDP‐43 encephalopathy neuropathologic changes (LATE‐NC) and tauopathies such as corticobasal degeneration (CBD), Pick's disease, progressive supranuclear palsy (PSP), or other subtypes of frontotemporal lobar degeneration with pTau inclusions (FTLD‐tau). Inclusion in Cohort 1 also required availability of Mini‐Mental State Examination (MMSE) scores obtained within 2 years of death, ensuring reliable clinicopathological correlation. Cohort 2 (*n* = 329) served as the primary test subsample, selected using similar exclusion criteria and temporal constraints to Cohort 1, and similarly limited to cases with MMSE data within 2 years of death. However, unlike Cohort 1, Cohort 2 did not exclude cases with comorbid pathology (DLB, LATE‐NC, FTLD‐tau, etc.). Cohort 3 (*n* = 58) included approximately 12 cases per Braak NFT stages (II–VI) that were randomly sampled to ensure balanced representation across disease stages. This third cohort was used to assess detection methods across anatomical regions and in varying histological stains. Together, these cohorts enabled systematic training, validation, and between‐center comparison of digital neuropathological assessment paradigms.

### 2.2 Tissue preparation and immunohistochemistry

Immunohistochemistry and analysis for this study was completed on four neocortical regions: middle frontal gyrus (MFG, Brodmann area [BA] 9), superior and middle temporal gyri (SMTG, BA 21/22), inferior parietal lobule (BA 40), and occipital striate cortex (BA 17/18/19). These regions of neocortex were routinely sampled at autopsy. Immunohistochemistry for pTau was performed on 8 μm‐thick sections from paraffin‐embedded human autopsy brain sections using antibodies against PHF‐1 antibody (mouse monoclonal, 1:1000, courtesy of the late Dr. Peter Davies) and AT8 (1:1000, Thermo Fisher Scientific Cat# MN1020, RRID: AB_223647). Slides were first deparaffinized in xylene and rehydrated through a graded ethanol series (100%, 95%, and 80%) to distilled water. Antigen retrieval was conducted in Dako Target Retrieval Solution, low pH (TRS Lo, pH 6; Cat. #GV805) at 95°C for 6 min, followed by a 10‐min cooldown at room temperature. Slides were rinsed in running distilled water for 10 min and equilibrated in 1× TBS on a shaker for 5 min. Endogenous peroxidase activity was quenched by incubation in a solution of 20% methanol and 3% hydrogen peroxide for 30 min. Following an additional rinse in distilled water and a 5‐min TBS wash, sections were blocked in 5% non‐fat dry milk prepared in 1× TBS for 1 h at room temperature. Primary antibody incubation was carried out overnight at 4°C in a humidified chamber. The following day, sections were washed twice in 1× TBS (5 min each) and incubated for 1 h at room temperature with a biotinylated horse anti‐mouse secondary antibody (Vector Laboratories, Cat #BA‐2000, 1:250). After two additional TBS washes, slides were treated for 1 h with the VECTASTAIN® Elite ABC Peroxidase kit (Vector Laboratories, Cat #PK‐6100), prepared according to manufacturer instructions. Visualization was achieved using DAB chromogen solution (DAKO, Cat #K3468), applied dropwise until adequate signal development was observed. Slides were then immersed in deionized water to halt the reaction, washed for 5 min, and counterstained with IMCB Harris Hematoxylin (#HH‐160). Following another rinse in deionized water, slides were dehydrated through ascending ethanol concentrations (80%, 95%, and 100%), cleared in xylene, and coverslipped using a permanent mounting medium. Slides were cured for 48 h prior to WSI scanning. Following staining, WSIs were digitized using a Leica/Aperio AT‐2 Scanner at 40× magnification.

At the UK‐ADRC, Braak NFT staging has historically been diagnosed using methodology grounded in evolving consensus‐based guidelines. The current study focused on Braak NFT stages IV through VI, which have traditionally been differentiated at the UK‐ADRC based on the density of pTau NFTs in the neocortex, identified using the PHF‐1 pTau antibody. Although consensus‐based methods focus on the striate and temporal lobe neocortices for differentiating severe Braak NFT stages, staining in the MFG and parietal cortex has also factored into the diagnostic thresholding rubric at the UK‐ADRC, although there was not a prior specific cutoff to differentiate Braak NFT stages IV or V [2, 4, 5, 6, 7, 8, 78, 79, 80].

### 2.3 Analysis pipeline using Aperio software

As described in detail previously [22], the Leica/Aperio “Genie” image analysis software system (referred to here as “Aperio”) was used to assess pTau pathology within selected grey matter regions of interest (ROIs). Full‐slide quantification was initially avoided because of excessive variability in grey‐to‐white matter ratios across cases and prohibitive processing time; rather, fixed‐size square ROIs (10 ROIs per slide, each 4 mm^2^) were placed throughout grey matter areas that displayed consistent pathological signal intensity [22] (Figure S1A, Supporting Information). ROIs were distributed across the tissue to maximize spatial separation while avoiding overlapping and regions with suboptimal staining or structural distortion. Non‐neural elements such as tissue folds, processing debris, and meningeal vasculature were manually excluded from the analysis using negative selection tools (Figure S1A). ROIs were manually placed according to a sampling strategy rather than by true random selection. This approach allowed ROIs to be distributed broadly and consistently across the available grey matter to enhance reproducibility, while avoiding staining artifacts and tissue distortion that could confound quantitative analysis.

Quantification of NFT density using the Aperio software was then conducted using a modified nuclear detection algorithm optimized for pTau (PHF‐1 antibody) immunoreactivity. A systematic evaluation of minimum nuclear size thresholds was used to minimize the inclusion of non‐NFT elements, such as glial nuclei or background signal, while preserving true positive detection. To generate a widely applicable method of analysis, multiple threshold conditions were tested across a representative case subset, and algorithmic outputs were compared to manual annotations to identify the optimal detection threshold. Final analyses were performed across the grey matter ROIs, with NFT density calculated as the total number of detected NFTs normalized to total analyzed tissue area (Figure S1C). Additionally, pTau burden was assessed using a customized Positive Pixel Count (PPC) algorithm tuned for chromogenic detection. Output values were expressed as the proportion of high‐intensity pixels relative to the total tissue area analyzed [22] (Figure S1D).

### 2.4 Updated analysis pipeline using HALO software

A revised analytical pipeline was developed using the HALO image analysis platform (version 4.1.6, Indica Labs, Albuquerque, NM). This updated workflow integrated three classifier modules into a single automated pipeline, allowing for streamlined preprocessing and improved tissue classification. The digital pipeline used previously stained and digitized WSIs and began by removing glass/background and meningeal tissue. The tissue detection classifier was tuned using a resolution setting of 2.0 μm/pixel and gated to only detect objects with a minimum size of 500 μm^2^. Training for the tissue detection classifier was conducted on >500 objects and 400,000 training iterations achieving an entropy score of <0.01. Next, a quality control classifier eliminated tissue folds, tears, areas of poor focus, and extraneous debris from usable tissue. The quality control classifier was similarly tuned using 2.0 μm/pixel resolution and set to detect objects >100 μm^2^. This classifier was also trained on >500 objects and completed 300,000 training iterations until its entropy value was <0.01. Finally, the grey/white matter segmentation classifier was used to distinguish cortical regions and generate distinct annotation layers between grey and white matter to be used for regional analysis. This final classifier was tuned using 7.00 μm/pixel resolution to ignore small variations in grey/white matter staining and only detected objects >5000 μm^2^. This classifier was also trained using >500 objects and underwent 750,000 training iterations that continued after reaching an entropy value of <0.01. Classifier validation was conducted to test the accuracy and precision of the grey/white segmentation. Annotated regions were separated into a training set (80% of annotated regions), validation set (10% of annotated regions), and test set (10% of annotated regions). Classifier validation achieved a precision score of 0.969, a recall score of 0.961 and an F1 score of 0.965 (Figure S2).

Following the automated detection pipeline, quantification of NFTs was first performed using the Object Colocalization module. This module detects and counts discrete immune‐positive objects based on thresholded chromogen intensity, spatial morphology, and size constraints. This algorithm allowed for refined identification of NFT structures throughout the entire segmented grey matter on each slide, with outputs expressed as density of NFTs per mm^2^. Parameters within the object colocalization analysis were optimized based on size, detection blur and contrast radii as well as contrast threshold and optical density, allowing for the exclusion of non‐specific signal and artifactual inclusions. The final HALO module configuration was optimized to accurately recognize and quantify the morphological signature of PHF‐1‐positive NFTs (i.e., counted NFTs, rather than just positive staining), while excluding dystrophic neurites and neuritic plaques.

pTau burden was further measured using the Area Quantification module, optimized to detect DAB‐labeled pixels and calculate the percentage of tissue surface area demonstrating immunopositivity (% of tissue stained). This approach provided high‐resolution surface‐based quantification of pTau pathology and was validated across the same anatomical regions previously analyzed with the Aperio system. However, as mentioned, unlike what was done in Aperio software, the HALO‐based analyses were conducted on the entire segmented grey matter on each slide. Quantitative outputs from both platforms were integrated with clinical metadata, including cognitive status (based on clinical consensus and final MMSE scores) and Braak NFT staging. This framework enabled comparison between the Aperio and HALO pipelines. The HALO object‐detection pipeline was configured to generate NFT‐specific quantification outputs in an attempt to emulate and update the Aperio‐derived NFT density measures, rather than broadly quantify all pTau‐positive elements. HALO also provided a complementary measure of overall pTau burden based on area quantification, thereby enabling like‐for‐like comparison with both Aperio NFT lesion density and positive‐pixel counts.

### 2.5 Clinicopathological correlation

To determine the optimal anatomical sampling and analytic strategy for modeling clinicopathological relationships in ADNC, we quantified both NFT density and overall pTau burden across four neocortical regions from each case. Using data derived from both the HALO and Aperio digital pathology platforms, we assessed the correlations between regional pathology and cognitive performance as measured by MMSE scores. For each metric, all possible combinations of one, two, three, and four regions were evaluated to identify sampling strategies that maximized predictive accuracy and efficiency. The strength of association between quantitative pathology and cognitive status was assessed using the coefficient of determination (*R* ^2^), with results visualized as heatmaps stratified by analysis platform and pathology type (Table S1).

### 2.6 Generation of data‐driven diagnostic staging thresholds

To further evaluate quantitative, clinically informed thresholds for severe‐stage Braak NFT staging classification, we focused on two neocortical regions, SMTG and occipital cortex. Using data from Cohort 1, NFT density (NFTs counted per mm^2^) was computed across both regions to generate a composite pTau pathology load for each case. Individuals were then rank ordered based on total NFT density, and cognitive status within 2 years of death was used to inform cut‐off selection. Thresholds were defined using criteria that combined both pathology and clinical impairment data. Braak NFT stage VI was restricted to individuals with a clinical diagnosis of dementia, irrespective of NFT burden. The threshold model derived from Cohort 1 was applied to Cohort 2, and cases were reassigned accordingly when pathology and cognitive data diverged from their originally diagnosed Braak NFT staging designation. This approach enabled data‐driven refinement of severe ADNC classification while maintaining continuity with the anatomical logic of the Braak NFT staging framework. Information depicting the change in Braak NFT staging status and average MMSE score in each Braak NFT stage can be found in Tables S1 and S2.

### 2.7 Data analyses

All statistical analyses were performed using either JMP Pro software version 14.0 (SAS institute, Cary, NC) or GraphPad Prism software version 8.0. GraphPad Prism was also used to generate all graphs and heatmaps.

## 3 RESULTS

### 3.1 Overall workflow and study design

Study goals and workflow are illustrated in Table 1 and Figure 1.

**TABLE 1:** Summary of the overarching goals of this study, including the methodological strategies employed and the corresponding tissue cohorts utilized for each objective.

Goal	Specific subgoal	Approach to accomplish goal	Cohort(s) used
Overarching goal	Optimize routine assessment of the key severe ADNC parameter (neocortical neurofibrillary pathology) using digital pathology to inform Braak neurofibrillary pathology staging	Establish a reproducible digital framework for quantitative pTau pathology measurement to enhance analytic precision and strengthen clinicopathological staging alignment	Cohorts 1/2
Clinical relevance of routine assessment	(a) Evaluate use of pTau burden vs. NFT density counts in terms of clinical relevance	Assess predictive validity of area‐based versus object‐based pTau metrics in relation to cognitive and functional outcomes	Cohorts 1/2
	(b) Evaluate impact of sampling (anatomical regions included in analyses) in terms of clinical relevance	Model the contribution of regional sampling strategies to disease‐stage discrimination and cognitive decline prediction	Cohort 1
	(c) Generate actual cutoffs for Braak NFT stages IV–VI	Derive quantitative thresholds for late‐stage Braak classification using data‐driven correlations between digital NFT metrics and clinical phenotype	Cohort 1
Standardization of routine assessment	(a) Assess the potential to change from Aperio‐based to HALO‐based digital segmentation, pathology recognition, and quantification	Perform cross‐platform validation to quantify inter‐system concordance and identify algorithmic or classifier‐based biases	Cohorts 1/2
	(b) Evaluate using same HALO modules at different research centers	Conduct multi‐center reproducibility analyses to define inter‐laboratory variance and optimize module harmonization	Cohort 3
	(c) Evaluate impact of (and methodologic strategies for) using different pTau antibodies	Compare digital quantification outputs across distinct pTau epitopes (AT8, PHF‐1) to characterize antibody‐specific signal variability	Cohort 3

**FIGURE 1:**
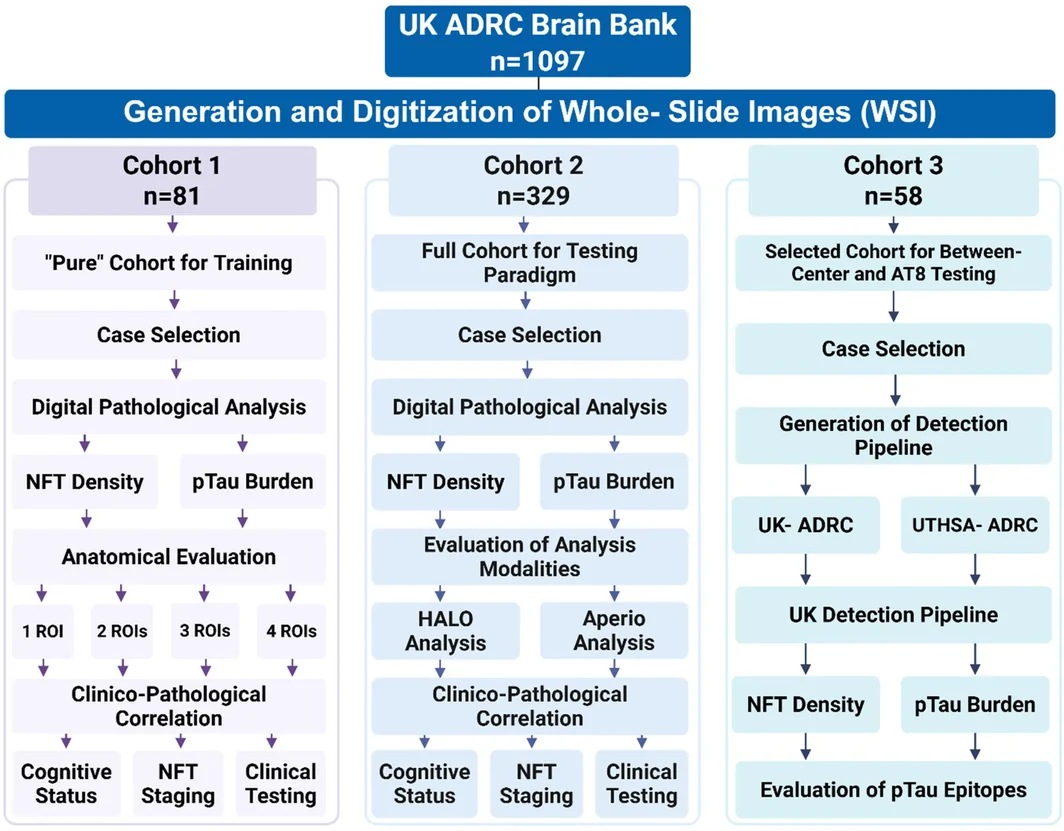
Analytical workflow for digital quantification of pTau pathology. Schematic overview of the study design integrating three sub‐cohorts from the UK‐ADRC Brain Bank used for the generation and digitization of whole‐slide images (WSI) used in quantitative tau pathology analysis. Cohort 1, comprising cases without severe comorbid pathologies and an MMSE score with 2 years of death (*n* = 81), served as a training set for the development of digital pathological analysis pipelines. Quantification of pTau density and burden across anatomically defined regions of interest was followed by clinicopathological correlation. Cohort 2 (*n* = 329), comprising all UK‐ADRC cases between 2011 and 2025 that met inclusion criteria, represents the primary testing cohort used to evaluate analytical performance across two digital platforms (HALO and Aperio) and were the basis for clinicopathological correlation analyses. Cohort 3 (*n* = 58) was used for inter‐laboratory validation, i.e., cross‐center reproducibility, as well as antibody comparability (AT8 and PHF‐1), with detection pipelines standardized and deployed across UK and UTHSA ADRCs.

Three autopsy cohort subsamples were used to evaluate digital quantification of pTau pathology across different stages of ADNC. In all three cohorts, participants spanned the full range of ADNC severity including Braak NFT stages 0–VI. Cognitive performance, as assessed by final MMSE scores, declined progressively with increasing neuropathological severity as expected. The Apolipoprotein E (*APOE*) genotype distribution was predominantly ε3/ε3, although the ε4 allele was more frequent in individuals with higher Braak NFT stages. Also as expected, comorbid neuropathological findings, including cerebrovascular disease (CVD) and hippocampal sclerosis of aging (HS‐A), were more frequent in the later stages of cognitive impairment. Postmortem intervals (PMI) were relatively consistent across groups, typically ranging from 2 to 8 h (Tables 2, 3, 4).

**TABLE 2:** Clinicopathological summary of “pure” ADNC cases used as training set (Cohort 1).

Cohort 1, *n* = 81									
Braak NFT stage	Sample size	Age at death (Avg.)	Other tauopathies^a^	LATE‐NC present	HS‐aging	CVD	Most common *APOE* genotype	MMSE (Avg.)	PMI, h (Avg.)
0	*n* = 2	78	0/2	0/2	0/2	1/2	ε2/ε3	30	6.0
	M(2) F(0)						1/2		
I	*n* = 19	84	0/19	0/19	0/19	6/19	ε3/ε3	29	4.8
	M(6) F(13)						13/19		
II	*n* = 20	84	0/20	0/20	0/20	6/20	ε3/ε3	29	5.7
	M(11) F(9)						12/20		
III	*n* = 5	86	0/5	0/5	0/5	1/5	ε4/ε4	28	5.6
	M(2) F(3)						4/5		
IV	*n* = 4	88	0/4	0/4	0/4	2/4	ε4/ε4	25	3.3
	M(3) F(1)						2/4		
V	*n* = 10	84	0/10	0/10	0/10	7/10	ε3/ε3	25	7.4
	M(6) F(4)						6/10		
VI	*n* = 10	71	0/10	0/10	0/10	3/10	ε4/ε4	11	10.0
	M(5) F(5)						7/10		
N/A	*n* = 11	80	4/11	0/11	0/11	1/11	ε3/ε3	26	7.76
	M(4) F(7)						5/11		

Abbreviations: CVD, cerebrovascular disease pathology; HS, aging: hippocampal sclerosis of aging; LATE‐NC, Limbic‐predominant age‐related TDP‐43 encephalopathy neuropathologic change; MMSE, minimental state examination; PMI, post‐mortem interval.

^a^ Other tauopathies include non‐ADNC conditions with tau proteinopathy such as Pick's disease, other subtypes of FTLD‐tau, etc. N/A cases omitted from Braak NFT staging analysis because of missing in un‐stageable Braak NFT stage, or the present of Lewy Body pathology.

**TABLE 3:** Expanded ADNC dataset including comorbid pathology (Cohort 2).

Cohort 2; *n* = 329									
Braak NFT stage	Sample size	Age at death (Avg.)	Other tauopathies^a^	LATE‐NC present	HS‐aging	CVD	Most common *APOE* genotype	MMSE (Avg.)	PMI, h (Avg.)
0	*n* = 6	82	0/6	0/6	0/6	4/6	ε3/ε3	26	6.3
	M(5) F(1)						3/6		
I	*n* = 50	82	0/50	4/50	3/50	17/50	ε3/ε3	27	6.5
	M(21) F(29)						31/50		
II	*n* = 55	88	0/55	5/55	8/55	22/50	ε3/ε3	25	6.7
	M(30) F(25)						35/55		
III	*n* = 28	88	0/28	6/28	3/28	14/28	ε3/ε3	25	5.6
	M(11) F(17)						15/28		
IV	*n* = 20	88	0/20	1/20	1/20	13/20	ε3/ε3	23	7.2
	M(11) F(9)						10/20		
V	*n* = 92	86	0/92	19/92	19/92	45/92	ε4/ε4	18	6.8
	M(54) F(38)						37/92		
VI	*n* = 53	77	0/53	8/53	11/53	21/53	ε4/ε4	10	9.2
	M(27) F(26)						32/53		
N/A	*n* = 25	77	7/25	1/25	1/25	4/25	ε3/ε3	26	8.8
	M(10) F(15)						8/25		

Abbreviations: CVD, cerebrovascular disease pathology; HS‐aging, hippocampal sclerosis of aging; LATE‐NC, Limbic‐predominant age‐related TDP‐43 encephalopathy neuropathologic change; MMSE, minimental state examination; PMI, post‐mortem interval.

^a^ Other tauopathies include non‐ADNC conditions with tau proteinopathy such as Pick's disease, other subtypes of FTLD‐tau, etc. Cases omitted from table represent cases with missing or unstageable Braak status. N/A cases omitted from Braak NFT staging analysis because of missing in un‐stageable Braak NFT stage, or the present of Lewy Body pathology.

**TABLE 4:** Cohort for cross‐center standardization testing (Cohort 3).

Cohort 3; *n* = 58									
Braak NFT stage	Sample size	Age at death (Avg.)	Other tauopathies^a^	LATE‐NC present	HS‐aging	CVD	Most common *APOE* genotype	MMSE (Avg.)	PMI, h (Avg.)
0	‐	‐	‐	‐	‐	‐	‐	‐	‐
I									
II	*n* = 12	88.4	0/12	8/12	3/12	6/12	ε3/ε3	24	6.2
	M(6) F(6)						10/12		
III	*n* = 11	87.8	0/11	5/11	1/11	8/11	ε3/ε3	24.5	5.4
	M(6) F(5)						4/11		
IV	*n* = 11	87.3	0/11	5/11	4/11	7/11	ε3/ε3	23.2	15.3
	M(7) F(4)						7/11		
V	*n* = 12	81.8	0/12	5/12	5/12	3/12	ε3/ε3	17.2	11.5
	M(7) F(5)						5/12		
VI	*n* = 12	79.4	0/12	9/12	4/12	6/12	ε4/ε4	6	6.8
	M(5) F(7)						7/12		

Abbreviations: CVD, cerebrovascular disease pathology; HS‐aging, hippocampal sclerosis of aging; LATE‐NC, Limbic‐predominant age‐related TDP‐43 encephalopathy neuropathologic change; MMSE, minimental state examination; PMI, post‐mortem interval.

^a^ Other tauopathies include non‐ADNC conditions with tau proteinopathy such as Pick's disease, other subtypes of FTLD‐tau, etc.

### 3.2 Digital quantification of pTau pathology across four neocortical regions

To establish a standardized digital neuropathology workflow, we implemented parallel quantification pipelines using two commercially available platforms, HALO and Aperio, applied to sections immunostained for PHF‐1. The updated pipeline generated two orthogonal quantitative outputs: NFT density (NFTs counted per mm^2^), based on object‐level detection, and pTau burden, reflecting the percentage of cortical grey matter positive for pTau via pixel‐based classification (% of tissue stained). These analyses were performed on four different neocortical regions implicated in ADNC, including MFG, SMTG, parietal cortex, and occipital cortex. AI‐driven workflows yielded segmentation of grey matter regions and successfully captured regional pathology patterns consistent with known disease topography (Figure 2).

**FIGURE 2:**
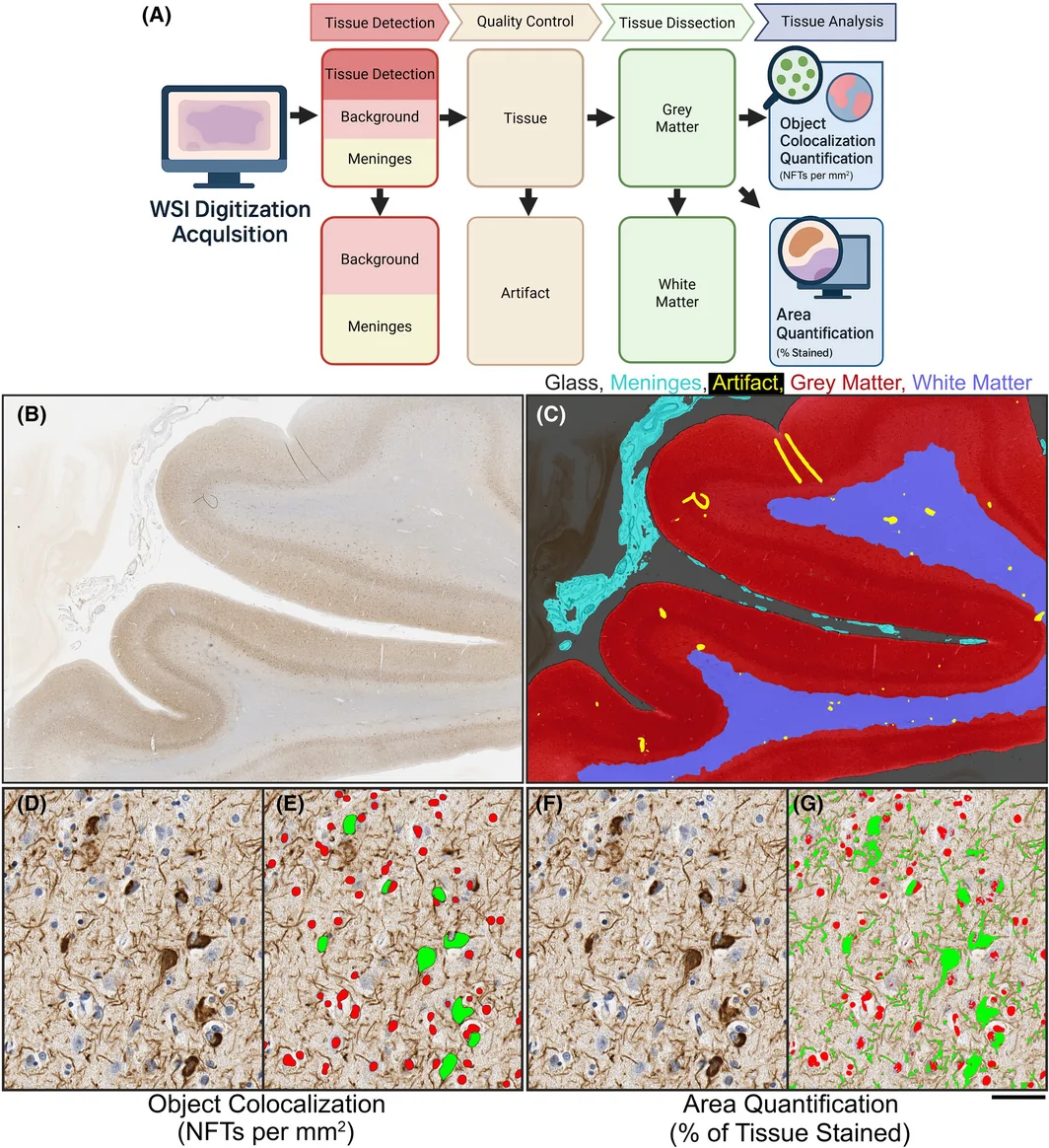
Workflow for digital whole‐slide image (WSI) processing and quantitative analysis of pTau pathology. (A) Diagram of the WSI processing pipeline depicting sequential stages of tissue detection, quality control, dissection, and quantitative analysis. Beginning with a fully digitized WSI immunostained for pTau (B), tissue sections show pseudo‐colored segmentation for regional delineation and quantification (C). AI detection delineated and omitted slide glass (background, black), meninges (cyan), and processing artifacts (yellow), were excluded prior to segmentation of gray matter (red) and white matter (blue) regions for computational assessment. Examples of automated detection algorithms used for object colocalization (NFT density, expressed as NFTs per mm^2^) (D–E; image markup shows detected NFTs pseudo‐colored green, cell nuclei pseudo‐colored red) and area‐based quantification (percentage of tissue stained) (F–G; image markup shows detected pTau immunopositivity false‐colored green, cell nuclei false‐colored red). Together, these steps define a reproducible digital pathology workflow for quantitative assessment of pTau pathology. Scale bar for panels D&G = 50 μm.

Using these segmentation methods, we assessed how well regionally sampled pTau pathology predicted cognitive status. For each case in Cohort 1, we analyzed the relationship (*R* ^2^) between final MMSE score and both NFT density and pTau burden derived from HALO and Aperio platforms (Figure 3A). All combinations of one, two, three, or four sampled neocortical regions were considered. As visualized in a heatmap of *R* ^2^ values, multiregional sampling provided stronger associations with cognitive impairment than single‐region sampling, with the most consistent performance observed when the SMTG and occipital cortex were combined (Figure 3A). Sampling limited to SMTG and occipital cortex yielded *R* ^2^ values comparable to full‐region models, consistent with their established relevance in Braak NFT staging [8]. These findings were stable across platforms and across quantification metrics (Figure 3B,C).

**FIGURE 3:**
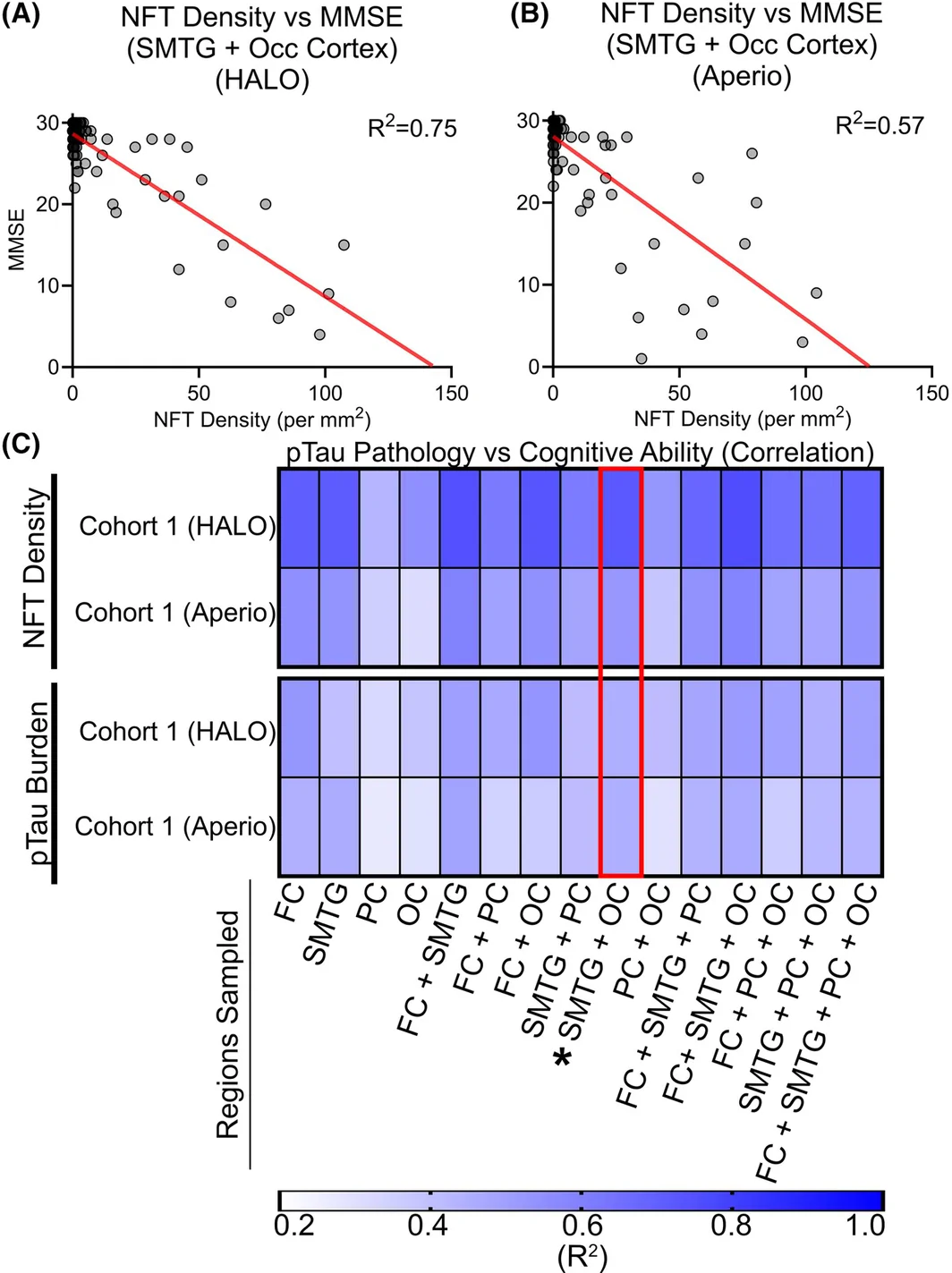
Results of different tissue sampling and analytic methodology for correlation between pTau pathology and clinical (final MMSE score) data. Representative scatterplots illustrate the relationship between NFT density in the SMTG and occipital cortex vs. final MMSE scores, quantified using HALO (A) and Aperio software (B). Heatmaps showing correlations (*R* ^2^) between cortical regions sampled for pTau burden or NFT density across analytical modalities (HALO and Aperio) within Cohort 1. Strong regional concordance was observed, with comparable performance between platforms. These findings demonstrate the robustness and reproducibility of the digital quantification pipeline across anatomical regions and analysis platforms (C). Note that NFT density shows stronger correlation with final MMSE scores than pTau burden, and HALO software produces stronger correlations between pTau pathology and final MMSE scores. FC, middle frontal gyrus (BA9); SMTG, superior and middle temporal gyri (BA 21/22); PC, parietal cortex (BA 40); OC, occipital cortex (BA 17/18/19). * (and red highlight) SMTG and occipital cortex sampling as recommended for Braak NFT staging in Alafuzoff et al. [8].

### 3.3 Quantitative NFT density stratified by clinical status to refine staging accuracy

After establishing that the use of NFT densities in two (SMTG and occipital) neocortical regions correlated with cognitive abilities, we proceeded to update the existing thresholds for each of the stages in severe ADNC. Rank‐ordering cases based on NFT densities revealed substantial inter‐individual variability across the cohort (Figure 4A). The highest NFT summed count among non‐impaired individuals was approximately 19 NFTs per mm^2^, while the impaired group extended well above 45 NFTs per mm^2^, establishing a potential data‐driven threshold criterion. We further examined how digital NFT quantification aligned with prior diagnosed Braak NFT staging (Figure 4B). Digital NFT counts were positively correlated with the original and Braak NFT classification (Figure 4C), with the updated model showing superior linearity (*R* ^2^ = 0.5140 vs. *R* ^2^ = 0.6582). Several cases were reassigned to higher or lower stages under the updated model, with final assignments better reflecting their quantitative burden (Figure 4C). These results suggest that digital pathology can enhance resolution and staging fidelity beyond traditional categorical assessments.

**FIGURE 4:**
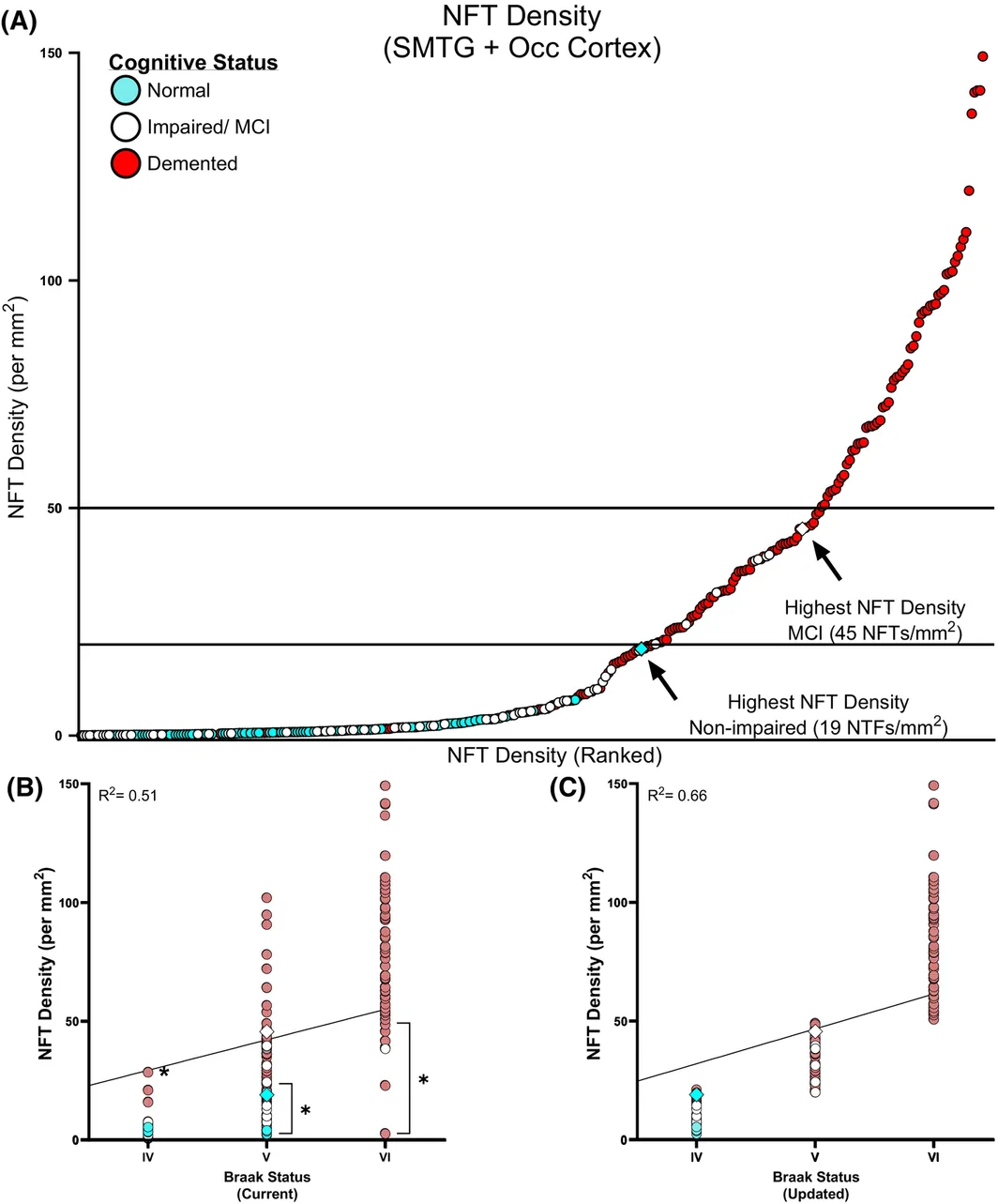
Quantitative distribution of NFT density and its association with Braak NFT stages. (A) Rank‐ordered NFT counts demonstrate substantial inter‐individual variability, with higher densities observed in cognitively impaired compared to non‐impaired individuals. Scatter plots reveal a strong positive correlation between digital NFT quantification and both current (B) and updated (C) Braak NFT staging schemes (*R* ^2^ = 0.5140 and *R* ^2^ = 0.6582, respectively), supporting the concordance between automated digital measures and traditional neuropathological staging of tau pathology. *Asterisks represent cases that were re‐classified in updated staging model.

### 3.4 Comparison of pTau detection between different digital pathology platforms

After establishing an updated workflow and Braak NFT stage classification, we next evaluated cross‐platform agreement. NFT density and pTau burden were independently quantified in each case and cortical region in Cohort 1 using both HALO and Aperio software. Cross‐platform comparisons demonstrated high linear correlation across all regions and metrics (*R* ^2^ = 0.91–0.98; Figure 5). This concordance confirmed that both object‐based and area‐based metrics are robust to methodological differences in platform architecture and algorithmic implementation.

**FIGURE 5:**
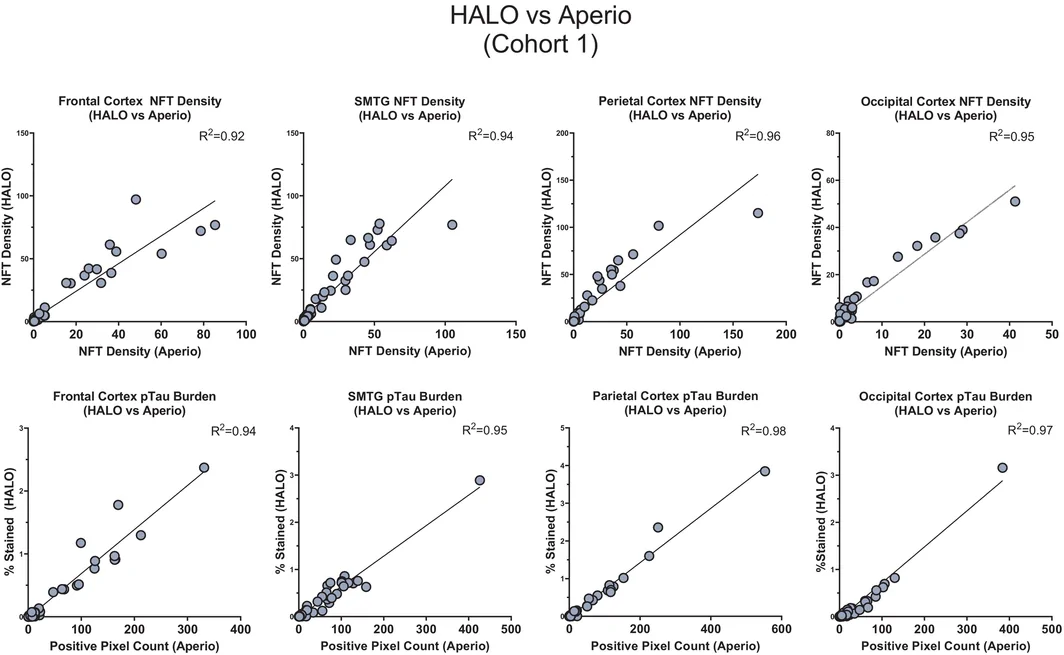
Correlation of digital quantification metrics between HALO and Aperio platforms in Cohort 1. Scatter plots demonstrate strong concordance between HALO and Aperio image analysis platforms for both NFT density (per mm^2^) (top row) and quantitative pTau burden (% grey matter area; bottom row) across four cortical regions: middle frontal gyrus (“Frontal”), superior middle temporal gyrus (“SMTG”), inferior parietal lobule (“Parietal”), and BA 17/18/19 (“Occipital”) cortices. Each data point represents an individual case from Cohort 1, consisting of “pure” Alzheimer's disease neuropathologic change (ADNC) cases without coexisting tauopathies or other impactful comorbidities, e.g., LATE‐NC. High coefficients of determination (*R* ^2^ = 0.9186–0.9831) indicate excellent agreement between the two digital pathology workflows, supporting cross‐platform reproducibility of pTau quantification.

Despite producing comparable quantitative metrics, HALO and Aperio workflows differed substantially in analytic workflows. As reported in Neltner et al., Aperio requires manual ROI placement and quality control, with semi‐automatic analysis steps [22]. For pTau analysis, a single slide can take up to 10 min for quality control and 30 min for pathology detection. In contrast, HALO utilizes a fully integrated AI pipeline that automates tissue detection, quality control, grey/white matter dissection, and analysis steps, often completing all stages in under 1 min per slide. These automation‐driven efficiencies dramatically reduce user burden while preserving analytic fidelity, making HALO particularly well‐suited for high‐throughput or multi‐site studies where standardization and scalability are essential (Table 5).

**TABLE 5:** Comparison of digital pathology workflow efficiency‐related parameters between HALO and Aperio platforms.

Classifier/analysis	Aperio	HALO
Tissue detection	10–30 s	4 s
	ROIs manually placed throughout tissue	Done automatically as part of AI pipeline
Quality control	5–10 min	17 s
	Done manually	Done automatically as part of AI pipeline
Tissue dissection	N/A	4 s
	N/A	Done automatically as part of AI pipeline
Object colocalization	~30 min	15–60 s
	Semi‐automatic; started standalone analysis	Done automatically as part of AI pipeline or as standalone analysis
Area quantification	~2 min	15–60 s
	Semi‐automatic; started standalone analysis	Done automatically as part of AI pipeline or as standalone analysis

Following development, training, and internal testing of the AI‐detection pipeline using Cohort 1, the automated pipeline was next applied to Cohort 2 to assess generalizability on a wider range of pathologies. As previously mentioned, Cohort 1 consisted of cases with “pure” ADNC pathology, while Cohort 2 encompassed a full spectrum of cases from the UK‐ADRC brain bank, including those with mixed and multiple pathologies. Application of the detection pipeline to Cohort 2 yielded similarly high correlation coefficients (*R* ^2^ = 0.78–0.90), confirming its ability to detect pTau pathology in more heterogeneous pathological cohorts that are also consistent with the findings generated within the ADRC since 2012. Together, these findings affirm the interchangeability of digital pathology metrics across software environments and support the feasibility of harmonized, efficient workflows for both research and clinical applications (Figure 6).

**FIGURE 6:**
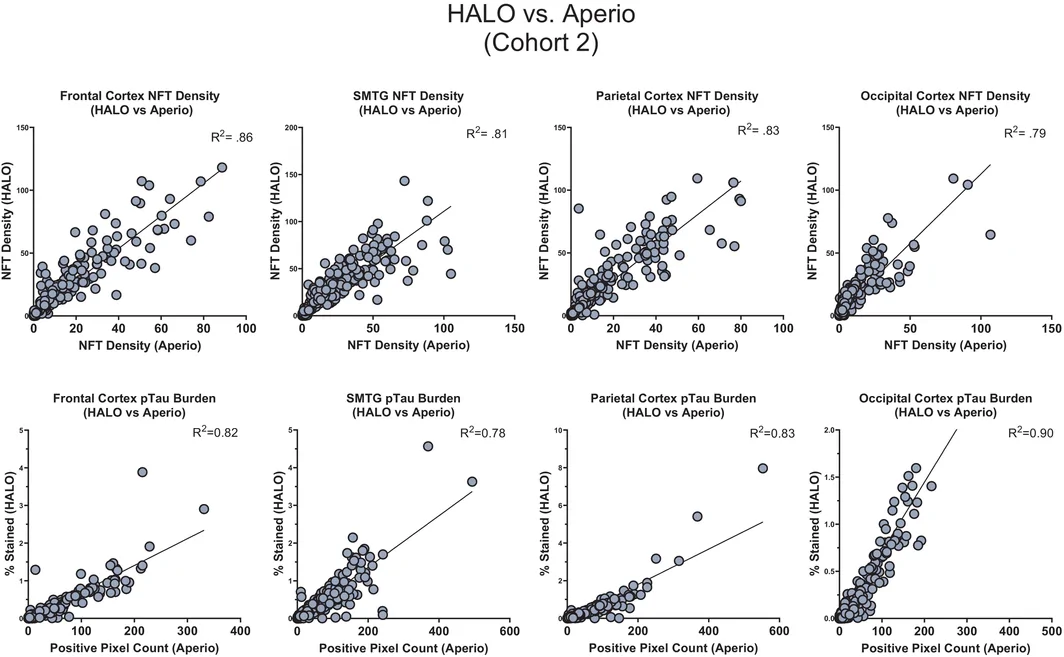
Comparative performance of HALO and Aperio digital analysis platforms in pTau pathology quantification (Cohort 2). Scatter plots comparing NFT density (NFTs per mm^2^) and pTau burden (positive pixel quantification or percent area stained) between HALO and Aperio analysis platforms across multiple cortical regions. Strong correlations for both metrics (*R* ^2^ = 0.65–0.86) confirm analytical consistency and reproducibility across independent digital pathology systems, supporting cross‐platform harmonization for quantitative assessment of tau pathology.

### 3.5 Assessment of pTau quantification across independent sites using harmonized pipelines

To determine whether digital quantification could be generalized and shared across different sites (i.e., researchers at different universities), identical imaging and analysis workflows were applied to matched cases from two independent brain banks (UK‐ADRC and UTHSA‐ADRC). NFT density and pTau burden were independently derived at each site for all four cortical regions. Inter‐site correlation coefficients exceeded *R* ^2^ = 0.99 across all comparisons, reflecting near‐identical quantification between sites (Figure 7).

**FIGURE 7:**
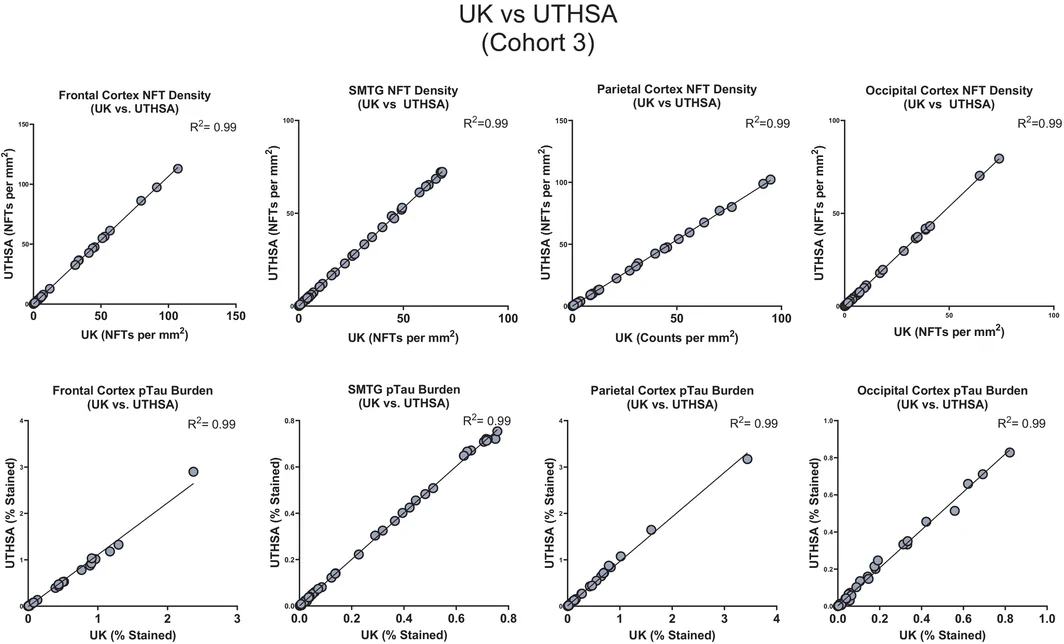
Cross‐center reproducibility of digital tau quantification between UK and UTHSA ADRCs (Cohort 3). Scatter plots comparing NFT density (per mm^2^) and pTau burden (percent area stained) across corresponding cortical regions between the UK and UTHSA ADRCs. Extremely high concordance (*R* ^2^ > 0.99 across all regions) demonstrates near‐identical performance between laboratories using harmonized image acquisition and analysis pipelines, confirming the reproducibility of digital pTau quantification across centers.

### 3.6 Assessment of pTau pathologic quantification across different pTau antibodies (PHF‐1 and AT8)

Lastly, we examined whether digital quantification is preserved across pTau epitopes by comparing pathology metrics from adjacent sections stained with pTau antibodies PHF‐1 and AT8 in Cohort 3. Strong correlations were observed across both NFT density and pTau burden in all four cortical regions (*R* ^2^ = 0.73–0.91; Figure 8), with particularly high agreement in SMTG and MFG. This validation is highly relevant given that the AT8 antibody is one of the most widely used commercially available pTau antibodies in both research and clinical applications [81, 82].

**FIGURE 8:**
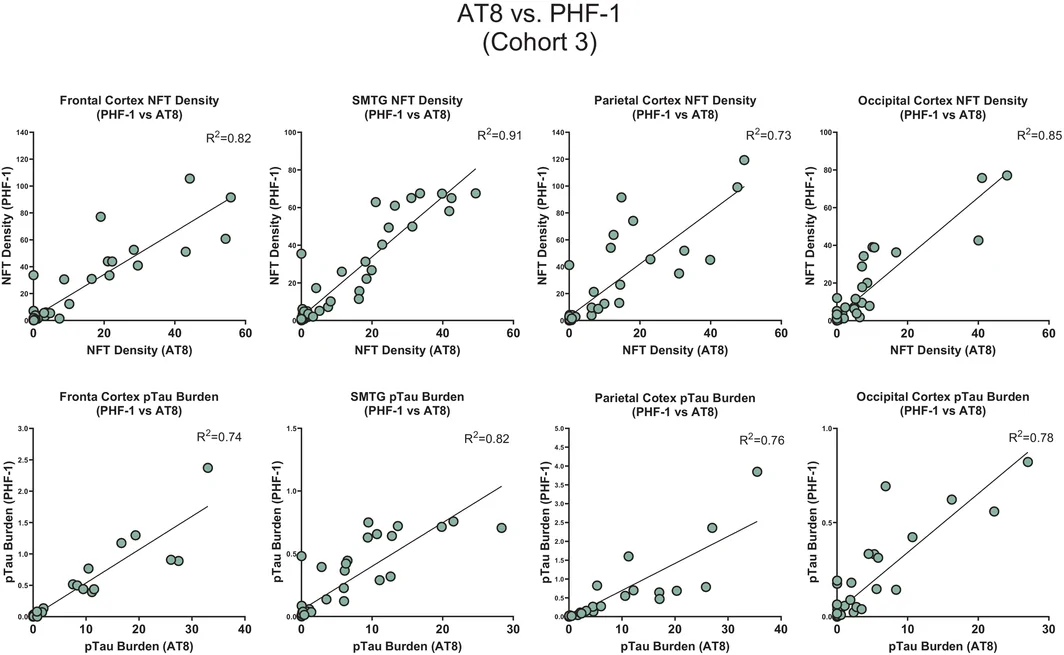
Comparative analysis of phospho‐tau antibodies AT8 and PHF‐1 for quantitative assessment of pTau pathology (Cohort 3). Correlation analyses of NFT density and pTau burden derived from sections stained with AT8 and PHF‐1 antibodies demonstrate strong agreement across cortical regions (*R* ^2^ = 0.73–0.91). Both antibodies yielded consistent quantitative measures of pTau pathology, validating methodological interchangeability for phosphorylated tau detection in digital neuropathological studies.

## 4 DISCUSSION

This study aimed to develop a data‐grounded framework for classifying Braak NFT stages IV–VI by integrating high‐resolution digital pathology, AI, and clinical metrics to support standardized diagnosis of severe ADNC. Through automated WSI analysis and cross‐platform validation, we generated quantitative measures of pTau burden and NFT density, both of which showed strong associations with antemortem cognitive status. These measures enabled a clinically informed distinction among higher Braak NFT stages [4, 5, 6, 7, 83]. Although the overall focus of this project was severe ADNC (Braak NFT stages IV–VI), the samples used (specifically those in Cohort 2) were not limited to cognitively impaired individuals. More specifically, 24% of the overall cohort were cognitively normal before death. The present approach thus provided a continuous, quantitative measure of pTau burden and NFT density that may help refine disease classification across the disease spectrum while preserving the established anatomical framework of Braak NFT staging. Importantly, these findings do not propose a modification of the Braak NFT staging system per se, but rather offer a reproducible, clinically relevant supporting protocol that complements the existing framework and enhances its clinical and research applicability. Moreover, the ability to apply clinical data‐informed, quantitative thresholds to pTau pathology facilitates improved diagnostic stratification, particularly in cases where semi‐quantitative staging yields incongruent clinical interpretations. These thresholds should be interpreted as data‐driven, retrospective refinements intended to harmonize classification within severe Braak NFT stages and are designed to provide a clinically informed quantitative framework that complements traditional Braak NFT staging by improving resolution and reproducibility in late‐stage ADNC. In such settings, digitally derived metrics provide a more data‐driven foundation for evaluating disease severity.

In the current study, the optimal anatomical regions sampled that we recommended coincided with BrainNet Braak staging as detailed by Alafuzoff et al. [8, 84]. Thus, quantification of pTau pathology from the SMTG and occipital cortex provided robust clinicopathological associations [4, 5, 6, 7, 83]. These regions are already included in standard ADRC sampling protocols, ensuring that no additional tissue acquisition or specialized processing is required [79]. This alignment with established workflows facilitates integration of the proposed digital metrics into routine neuropathologic assessment and multicenter research efforts.

Our updated approach proposes quantitative cutoffs for categorization of advanced stages of pTau pathology in ADNC, based on AI‐derived metrics integrating pathological and cognitive data. While recent studies have demonstrated the utility of HALO and QuPath‐based digital quantification of pTau pathology, the present study specifically emphasizes the cross‐correlation of digital pathology and clinical data with Braak NFT staging [33, 85, 86, 87]. An underlying assumption for the current study is that the pathological grading of disease should reflect clinical outcomes. More specifically, the most severe disease grade should reliably reflect clinical impairment. While this generally holds true for ADNC, the criteria for underlying pathologic staging remain open to refinement (see, e.g., References [3, 78]).

While Braak NFT staging remains a foundational system for mapping the hierarchical progression of pTau pathology, it is constrained by its ordinal structure, interpretive variability, and imperfect correlation with clinical outcomes [88, 89, 90]. By integrating DL‐based quantification with longitudinal cognitive measures, our analyses improved these gaps. This combined approach enhances clinical‐pathological correlations and reduces variability inherent in categorical assessments, particularly in the context of late‐stage disease.

A key strength of this study lies in its reproducibility and scalability across institutions. Historically, inter‐laboratory variation in antibody selection and regional sampling have hindered comparability in ADNC staging. In addition to methodological inconsistencies across institutions, significant variability also arises from differences in the choice of pTau antibodies used for immunohistochemistry. However, advances in whole‐slide imaging and AI‐based tissue segmentation now permit direct sharing of classifier models and quantification parameters, an achievement that until recently was technically prohibitive. The use of commercially available digital pathology platforms, standardized classifier architectures, and easily shared analytical pipelines allows for standardized replication of quantitative outputs between centers, as demonstrated by strong data concordance across sites and staining protocols. However, the data also highlighted the shortcomings in the clinical‐pathological correlation of our own archival Braak NFT staging data. Nevertheless, this study provides proof of concept that standardized, AI‐based detection pipelines can be readily deployed and implemented in external settings across digital platforms. Once trained on tissue from a single site, these pipelines can be distributed electronically, via direct file transfer or public repositories such as Github, and used without the need for retraining or site‐specific optimization, supporting the goal of broader adaptation of harmonized analytical frameworks in neuropathology.

Braak NFT staging has served as the principal framework for indexing the progression of pTau neurofibrillary pathology in amnestic dementia. However, recent multi‐center evaluations, most notably from the BrainNet Europe Consortium, have revealed considerable interpretive variability in staging outcomes across institutions, even when guided by consensus protocols [68, 84, 91]. The protocol that could be regarded as the “gold standard” for Braak NFT staging, proposed by Alafuzoff et al. (with Dr. Heiko Braak a coauthor), assigned stages IV–VI based on the distribution of pTau pathology across defined neocortical regions (occipital/striate cortex and temporal neocortex). This system operationalizes “+++,” “++,” “+,” or “−” severity scoring of AT8‐immunoreactive neuropil threads [8], thereby emphasizing a form of pTau pathology distinct from NFTs per se. In contrast, the 2012 NIA‐AA consensus ADNC diagnostic guidelines were notably less prescriptive, permitting the use of either silver impregnation staining or pTau immunohistochemistry to determine Braak NFT stages [2]. Taken together, these considerations underscore major practical limitation of the Braak NFT staging framework: its reliance on semi‐quantitative, anatomically bounded classification introduces substantial variability in staging fidelity, particularly in advanced stages where clinical implications are greatest. In response, there is a need for more reproducible, quantitative, and cognitive status‐informed approaches that augment traditional anatomical hierarchies with digital pathology and machine learning–based measurements of pTau pathology. Our study addressed these needs by augmenting categorical anatomical staging with continuous, regionally sampled, and clinically validated digital metrics that refine late‐stage classification while preserving conceptual continuity with the original Braak NFT staging framework.

This study also demonstrated that harmonized AI‐based pathology workflows can be effectively implemented across institutions, imaging platforms, and pTau antibodies. The ability to deploy classifier models and quantification parameters across brain banks and pathology centers represents a significant advance in reproducibility, enabling multi‐site studies of unprecedented scale. In the context of the ADRC network, such workflows facilitate standardization across autopsy cohorts and support large‐scale aggregation of digital pathology datasets. This work also underscores the feasibility of integrating AI‐derived metrics into formal neuropathological staging guidelines. As initiatives such as the NIA‐AA seek to refine ADNC diagnostic frameworks, incorporating quantitative, cognition‐informed metrics may help mitigate inter‐rater variability and strengthen the clinicopathological bridge. Our approach builds on the conceptual foundation of Braak NFT staging, providing greater resolution and improved clinical interpretability through consistent and transparent measurement.

Although both antibodies AT8 and PHF‐1 detect pathological pTau aggregates, the two antibodies bind to distinct tau phosphorylation epitopes and therefore label different molecular states of pTau pathology [81]. More specifically, AT8 recognizes phosphorylation at tau protein amino acid residues serine202/threonine205, which is commonly believed to be a marker of early conformational change and is abundant in pre‐NFT and neuropil thread pTau+ structures [80, 81, 82, 92, 93]. In contrast, PHF‐1 recognizes a phosphorylated epitope at tau protein amino acid residues serine396/serine404, typically associated with more mature NFTs [81, 82, 93, 94, 95, 96, 97]. These epitope‐specific affinities result in differences in staining patterns and signal intensity across brain regions and disease stages, which could influence digital detection algorithms trained on a single antibody type. As such, harmonizing outputs across cohorts and platforms requires careful validation of antibody‐specific classifiers and threshold parameters to ensure accurate and reproducible quantification of pTau pathology regardless of immunolabeling strategy. Our results indicated that the results of AT8 and PHF‐1 immunolabeling can be harmonized in a systematic way.

Some limitations of the current study warrant consideration. The MMSE test, while widely available and more granular than dichotomizing “dementia/no‐dementia” (or applying the 0–3 Clinical Dementia Rating scale), provides a still‐imperfect measure of global cognition that may ignore domain‐specific deficits more closely aligned with regional pTau burden [98, 99]. Future studies should include comprehensive neuropsychological batteries to better capture interesting clinicopathological relationships. Moreover, while digital readouts were generally consistent across PHF‐1 and AT8 antibodies, subtle differences in epitope binding and staining intensity may still introduce variability in cross‐site comparisons. Additionally, as WSIs included in this project incorporated nearly all cases in the UK‐ADRC biobank dating back to 2011, some variability in histological staining was unavoidable. Furthermore, our sampling focused on neocortical regions and did not include subcortical or allocortical areas, which may be critical for understanding atypical or early‐onset phenotypes [14, 80, 100, 101]. While commercial digital pathology tools such as HALO and Aperio software have enabled significant progress, they are not without their own shortcomings. They remain limited by licensing constraints, high costs, and historically restricted cross‐institutional compatibility. These barriers have prompted the development of federated, open‐access infrastructures such as the Brain Digital Slide Archive (BDSA; NIH grants U24 NS133945 and U24 NS133949), which fosters collaborative slide annotation, AI model training, and harmonized data sharing across institutions [1, 102]. Our integration with this initiative reflects the growing emphasis on open frameworks as essential infrastructure for scalable, reproducible, and equitable neuropathology. These efforts parallel successful initiatives in neuroimaging and genomics, where harmonized data repositories have catalyzed innovation in diagnostics and biomarker discovery.

This study contributes to ongoing efforts to modernize neuropathological assessment by advancing a scalable model for harmonized digital pathology that links pTau quantification with cognitive status. By enabling the deployment of AI‐driven pipelines across multiple sites and software platforms, our approach supports emerging standards for interoperable, clinically anchored analysis. Importantly, our findings validate that shared classifier modules and regionally optimized sampling can produce reproducible outputs that enhance disease classification, offering a step towards the implementation of multimodal staging systems. Within this evolving landscape, digital pathology is a key requirement for modern neuropathology, supporting precision phenotyping, cross‐disciplinary insight, and diagnostic fidelity. The present study contributes to these efforts by integrating digital histology with clinical data, reinforcing the field's movement toward holistic, standardized, and clinically interpretable assessments of neurodegenerative disease.

## 5 CONCLUSION

This study demonstrated that quantitative digital pathology, when integrated with clinical cognitive data, can enhance and rationalize Braak NFT staging of severe ADNC. By deploying harmonized AI‐driven pipelines across institutions, we derived reproducible metrics of pTau pathology that correlated more strongly with cognitive impairment and offered improved delineation of severe Braak NFT stages. These results underscore the feasibility of cognition‐informed digital staging and support a shift toward standardized, scalable approaches for neurodegenerative disease diagnosis.

## AUTHOR CONTRIBUTIONS

**Ryan K. Shahidehpour**: Conceived the work and primary writer; acquisition, analysis, and interpretation of data for the work; drafting the work for important intellectual content; final approval of the version to be published; agreement to be accountable for all aspects of the work. **Allison M. Neltner**: Acquisition, analysis, and interpretation of data for the work; drafting the work; final approval of the version to be published; agreement to be accountable for all aspects of the work. **Mitchell A. Klusty**: Acquisition, analysis, and interpretation of data for the work; drafting the work; final approval of the version to be published; agreement to be accountable for all aspects of the work. **Cole Corbett**: Acquisition, analysis, and interpretation of data for the work (from UTHSA aspect); drafting the work; final approval of the version to be published; agreement to be accountable for all aspects of the work. **Angelique D. Gonzalez**: Acquisition, analysis, and interpretation of data for the work (from UTHSA aspect); drafting the work; final approval of the version to be published; agreement to be accountable for all aspects of the work. **David A. Gutman**: Acquisition and interpretation of data for the work (from federated network aspect); drafting the work; final approval of the version to be published; agreement to be accountable for all aspects of the work. **David W. Fardo**: Acquisition, analysis (particularly statistical analyses), and interpretation of data for the work; drafting the work; final approval of the version to be published; agreement to be accountable for all aspects of the work. **Adam D. Bachstetter**: Acquisition, analysis (particularly image analyses), and interpretation of data for the work; drafting the work; final approval of the version to be published; agreement to be accountable for all aspects of the work. **Cody Bumgardner**: Acquisition, analysis, and interpretation of data for the work; drafting the work; final approval of the version to be published; agreement to be accountable for all aspects of the work. **Margaret E. Flanagan**: Acquisition, analysis, and administrative and regulatory aspects; interpretation of data for the work (from UTHSA aspect); drafting the work; final approval of the version to be published; agreement to be accountable for all aspects of the work. **Peter T. Nelson**: Conceived the work and was primary coauthorial editor; acquisition, analysis, and interpretation of data for the work; drafting the work for important intellectual content; final approval of the version to be published; agreement to be accountable for all aspects of the work; corresponded the manuscript.

## FUNDING INFORMATION

This work was supported by the National Institute on Aging (NIA) under grant numbers: T32 AG078110, P30 AG072946, U24 NS133945, U24 NS133949, and R01 AG054459.

## CONFLICT OF INTEREST STATEMENT

The authors declare no conflicts of interest.

## ETHICS STATEMENT

The University of Kentucky Institutional Review Board approved the use of autopsy material from the UK‐ADRC (UK IRB # 44009). Informed consent was obtained from UK‐ADRC research participants (and/or their caregivers) as stipulated in University of Kentucky IRB #44009.

## Supporting information


**Figure S1.** Quantification of pTau pathology using Aperio ImageScope software.
**Figure S2.** Representative workflow for whole‐slide image processing and tissue classification.
**Table S1.** Cognition‐informed reassignment of Braak NFT stages using digital pathology‐derived thresholds in Cohort 1.
**Table S2.** Cognition‐informed reassignment of Braak NFT stages using digital pathology‐derived thresholds.
**Table S3.** Matrix of *R* ^2^ values underlying the heatmap in Figure 3, showing strength of association between regional pTau pathology and MMSE scores across all sampled cortical regions.

## Data Availability

The data (including HALO modules) that support the findings of this study are available from the corresponding author.
