# Supplementary material for: Data‐driven thresholds for standardized classification of severe Alzheimer's disease neuropathology using digital neuropathology
Source: Brain Pathol. 2026 Apr 26;36(6):e70102. doi: 10.1111/bpa.70102 (PMC13630913; doi:10.1111/bpa.70102)
Supplement: Supplementary file 1 [file BPA-36-e70102-s001.docx]

**Supplemental Figure 1: Quantification of pTau pathology using Aperio ImageScope software.** This figure demonstrates the two primary methods of pTau quantification used in the UK-ADRC. (A) Low-magnification image showing two regions of interest (ROIs) placed within cortical grey matter for pTau quantification. (B) Higher-magnification view of one ROI demonstrating pTau immunostaining before analysis. (C) Detection of NFTs using the Aperio algorithm, showing automated color overlays identifying individual pTau-positive tangles (blue, yellow, orange based on detected optical density). (D) Positive pixel count analysis of the same ROI, where red pixels represent pTau-positive areas used for quantification. The inset highlights pixel-level classification of pTau burden. Photomicrographs originally captured at 40x magnification. Scale bars: (A) 1 mm; (D) 200 µm; insets, 50 µm.

**Supplemental Figure 2: Representative workflow for whole-slide image processing and tissue classification.** (A) After whole-slide images are digitized in entirety, there are first analyzed using an automated detection pipeline. (B) A tissue detection classifier is used to define tissue area and exclude background/non-tissue regions and meninges. (C) Tissue quality control is assessed to exclude staining artifacts and poor-quality staining. (D) Lastly, tissue segmentation is completed separating grey and white matter for downstream quantitative analysis. Photomicrographs captured at 1x magnification and scale bar represents 2mm.

**Supplemental Table 1: Cognition-informed reassignment of Braak NFT stages using digital pathology-derived thresholds in cohort 1**

| **Cohort 1; n=81** | | | | | | | | |
| --- | --- | --- | --- | --- | --- | --- | --- | --- |
| **Current Braak NFT Stage** | | | | **Updated Braak NFT Stage** | | | |  |
| **Braak NFT**  **stage** | **Sample size** | **Age at death (Avg.)** | **MMSE**  **(Avg.)** | **Braak NFT stage** | **Sample size** | **Age at death**  **(Avg.)** | **MMSE**  **(Avg.)** | **Difference in MMSE score** |
| 0 | n=2 | 78 | 30 | 0 | n=2 | 78 | 30 | 0 |
|  | M (2) F(0) |  |  |  | M(2) F(0) |  |  |  |
| I | n=19 | 84 | 29 | I | n=19 | 84 | 29 | 0 |
|  | M(6) F(13) |  |  |  | M(6) F(13) |  |  |  |
| II | n=20 | 84 | 29 | II | n=20 | 84 | 29 | 0 |
|  | M(11) F(9) |  |  |  | M(11) F(9) |  |  |  |
| III | n=5 | 86 | 28 | III | n=5 | 86 | 28 | 0 |
|  | M(2) F(3) |  |  |  | M(2) F(3) |  |  |  |
| IV | n=4 | 88 | 25 | IV | n=10 | 86 | 26 | 1 |
|  | M(3) F(1) |  |  |  | M(7) F(3) |  |  |  |
| V | n=10 | 84 | 25 | V | n=5 | 83 | 25 | 0 |
|  | M(6) F(4) |  |  |  | M(2) F(3) |  |  |  |
| VI | n=10 | 71 | 11 | VI | n=9 | 71 | 9 | -2 |
|  | M(5) F(5) |  |  |  | M(5) F(4) |  |  |  |
| N/A | n=11 | 80 | 26 | N/A | N/A | N/A | N/A | N/A |
|  | M(4) F(7) |  |  |  | N/A |  |  |  |

**Abbreviations:** MMSE: Minimental State Examination.

| **Cohort 2; n=329** | | | | | | | | |
| --- | --- | --- | --- | --- | --- | --- | --- | --- |
| **Current Braak NFT Stage** | | | | **Updated Braak NFT Stage** | | | |  |
| **Braak NFT**  **stage** | **Sample size** | **Age at death (Avg.)** | **MMSE**  **(Avg.)** | **Braak NFT stage** | **Sample size** | **Age at death**  **(Avg.)** | **MMSE**  **(Avg.)** | **Difference in MMSE score** |
| 0 | n=6 | 82 | 26 | 0 | n=6 | 82 | 26 | 0 |
|  | M(5) F(1) |  |  |  | M(5) F(1) |  |  |  |
| I | n=50 | 82 | 27 | I | n=50 | 82 | 27 | 0 |
|  | M(21) F(29) |  |  |  | M(21) F(29) |  |  |  |
| II | n=55 | 88 | 25 | II | n=55 | 88 | 25 | 0 |
|  | M(30) F(25) |  |  |  | M(30) F(25) |  |  |  |
| III | n=28 | 88 | 25 | III | n=28 | 88 | 25 | 0 |
|  | M(11) F(17) |  |  |  | M(11) F(17) |  |  |  |
| IV | n=20 | 88 | 23 | IV | n=54 | 88 | 21 | -2 |
|  | M(11) F(9) |  |  |  | M(32) F(22) |  |  |  |
| V | n=92 | 86 | 18 | V | n=59 | 84 | 16 | -2 |
|  | M(54) F(38) |  |  |  | M(28) F(31) |  |  |  |
| VI | n=53 | 77 | 10 | VI | n=56 | 78 | 9 | -1 |
|  | M(27) F(26) |  |  |  | M(34) F(22) |  |  |  |
| N/A | 25 | 77 | 26 | N/A | N/A | N/A | N/A | N/A |
|  | M(10) F(15) |  |  |  | N/A |  |  |  |

**Supplemental Table 2: Cognition-informed reassignment of Braak NFT stages using digital pathology-derived thresholds.**

**Abbreviations:** MMSE: Minimental State Examination.

**Supplemental Table 3: Matrix of R² values underlying the heatmap in Figure 3, showing strength of association between regional pTau pathology and MMSE scores across all sampled cortical regions.**

**Abbreviations:** Frontal cortex= F, SMTG= S, Parietal Cortex= P, Occipital Cortex= O

| **Cohort 1** | | | | | | | | | | | | | | | | |
| --- | --- | --- | --- | --- | --- | --- | --- | --- | --- | --- | --- | --- | --- | --- | --- | --- |
| **NFT Density** | | | | | | | | | | | | | | | | |
|  | **F** | **S** | | **P** | **O** | **F+S** | **F+P** | **F+O** | **S+P** | **S+O** | **P+O** | **F+S+P** | **F+S+O** | **F+P+O** | **S+P+O** | **F+S+P+O** |
| **NFT Density (HALO)** | 0.702 | 0.711 | | 0.432 | 0.549 | 0.744 | 0.615 | 0.730 | 0.613 | 0.750 | 0.527 | 0.675 | 0.752 | 0.646 | 0.640 | 0.689 |
| **NFT Density (Aperio)** | 0.549 | 0.534 | | 0.352 | 0.308 | 0.591 | 0.493 | 0.545 | 0.484 | 0.579 | 0.376 | 0.541 | 0.581 | 0.491 | 0.481 | 0.535 |
| **Tau Burden** | | | | | | | | | | | | | | | | |
|  | **F** | | **S** | **P** | **O** | **F+S** | **F+P** | **F+O** | **S+P** | **S+O** | **P+O** | **F+S+P** | **F+S+O** | **F+P+O** | **S+P+O** | **F+S+P+O** |
| **pTau Burden (HALO)** | 0.528 | | 0.397 | 0.317 | 0.379 | 0.499 | 0.467 | 0.531 | 0.409 | 0.450 | 0.408 | 0.478 | 0.515 | 0.494 | 0.446 | 0.495 |
| **pTau Burden (Aperio)** | 0.446 | | 0.458 | 0.270 | 0.295 | 0.490 | 0.340 | 0.360 | 0.408 | 0.450 | 0.291 | 0.441 | 0.466 | 0.355 | 0.417 | 0.437 |
